# Examination of Child and Adolescent Hospital Admission Rates in Queensland, Australia, 1995–2011: A Comparison of Coal Seam Gas, Coal Mining, and Rural Areas

**DOI:** 10.1007/s10995-018-2511-4

**Published:** 2018-03-02

**Authors:** Angela K. Werner, Kerrianne Watt, Cate Cameron, Sue Vink, Andrew Page, Paul Jagals

**Affiliations:** 1 Sustainable Minerals Institute, The University of Queensland, St. Lucia, QLD Australia; 2 College of Public Health, Medical and Veterinary Sciences, James Cook University, Townsville, Australia; 3 Jamieson Trauma Institute, Royal Brisbane & Women’s Hospital, Metro North Hospital and Health Services District, Brisbane, Australia; 4 School of Public Health and Social Work, Queensland University of Technology, Brisbane, Australia; 5 Centre for Health Research, Western Sydney University, Penrith, NSW Australia; 6 Children’s Health and Environment Programme, Centre for Children’s Health Research, University of Queensland, Brisbane, Australia; 7 Level 6, CWiMI, University of Queensland, Corner Staffhouse and College Roads, Sir James Foots Bldg (47a), St. Lucia, QLD 4072 Australia

**Keywords:** Adolescents, Children, Coal seam gas, Queensland, Unconventional natural gas

## Abstract

*Objectives* At present, coal seam gas (CSG) is the most common form of unconventional natural gas development occurring in Australia. Few studies have been conducted to explore the potential health impacts of CSG development on children and adolescents. This analysis presents age-specific hospitalisation rates for a child and adolescent cohort in three study areas in Queensland. *Methods* Three geographic areas were selected: a CSG area, a coal mining area, and a rural area with no mining activity. Changes in area-specific hospital admissions were investigated over the period 1995–2011 in a series of negative binomial regression analyses for 19 International Classification of Diseases (ICD) chapters, adjusting for sociodemographic factors. *Results* The strongest associations were found for respiratory diseases in 0–4 year olds (7% increase [95% CI 4%, 11%] and 6% increase [95% CI 2%, 10%] in the CSG area relative to the coal mining and rural areas, respectively) and 10–14 year olds (9% increase [95% CI 1%, 18%] and 11% increase [95% CI 1%, 21%] in the CSG area compared to the coal mining and rural areas, respectively). The largest effect size was for blood/immune diseases in 5–9 year olds in the CSG area (467% increase [95% CI 139%, 1244%]) compared to the rural area with no mining activity. *Conclusions* for *Practice* Higher rates of hospitalisation existed in the CSG area for certain ICD chapters and paediatric age groups, suggesting potential age-specific health impacts. This study provides insights on associations that should be explored further in terms of child and adolescent health.

## Significance

*What is already known on this subject?* A growing body of research exists on the potential health-related impacts of unconventional natural gas development. While children have been identified as a vulnerable group, there are few studies that have specifically examined child and adolescent health in relation to such development.

*What this study adds?* This study adds to the available evidence base by exploring child and adolescent health impacts related to coal seam gas development. The coal seam gas area did have higher rates of hospitalisation for certain health outcomes compared to coal mining and rural areas. This suggests potential health impacts that need to be examined further.

## Introduction

Coal seam gas (CSG) is a form of unconventional natural gas sourced from coal seams, with the gas being held in place by hydraulic pressure. Other forms of unconventional natural gas include shale gas and tight gas. CSG and shale gas are the most economically important unconventional natural gas resources. CSG is typically sourced from formations 300–1000 m deep while shale gas is sourced from formations 1000 m to > 2000 m deep (Ross and Darby 2013). Extraction of shale and tight gases requires hydraulic fracturing to release the gas from the source rock due to lower permeability, while CSG extraction may require hydraulic fracturing in some instances to increase permeability (Cook et al. 2013). Development of these different types of unconventional gas has been expanding worldwide, with major gas reserves located in a number of countries (Werner et al. 2015). Australia is no exception to this expansion.

At present, only CSG resources are being exploited in Australia, although shale gas and tight gas reserves do exist (Department of Natural Resources and Mines 2015). The CSG industry has been expanding throughout large areas of Queensland, Australia in recent years. The majority of Queensland’s CSG is sourced from the Bowen and Surat Basins (Department of Natural Resources and Mines 2014). CSG production began in the Bowen Basin in 1998, with the industry recognised as a stand-out sector since 2000 (Queensland Government 2011). Production began in the Surat Basin in 2005, adding to the rapid expansion of Queensland’s CSG industry (Queensland Government 2014). It is projected that there will be up to 40,000 producing wells in Queensland in the next few decades (Lacey and Lamont 2014; Measham and Fleming 2013).

Worldwide, there has been public unease about the potential health impacts of unconventional natural gas development (UNGD). Despite these concerns, a literature review on UNGD and health found that epidemiologic studies conducted in Australia (and elsewhere) examining the impact of CSG development on health are limited (Werner et al. 2015). In particular, no peer-reviewed studies in Australia have investigated the potential impacts of CSG development on children and adolescents. In the United States of America (USA), while studies are limited, some studies have examined the impacts of shale gas and tight gas development on children. While these studies can be referenced to better understand potential health impacts associated with CSG development, it is unclear how the conclusions translate to the CSG context (Vaneckova and Bambrick 2014). Local studies are needed as health effects vary with geology, demography, population proximity to UNGD, vulnerability, development techniques, and the companies and governing bodies involved (Bharadwaj and Goldstein 2015).

Research has shown that residents may be exposed to chemical and non-chemical stressors from UNGD, with exposures accumulating over time or as additional development occurs (Adgate et al. 2014). The major stressors that could affect health are air contamination, ground and surface water contamination, truck traffic, noise pollution, accidents and/or malfunctions, and psychosocial stress (Adgate et al. 2014). Exposure is likely to occur primarily through ingestion of contaminated drinking water or inhalation of air pollutants. Exposure to air pollutants, including particulate matter, has been linked to health outcomes such as respiratory and cardiovascular diseases, low birth weight, and preterm birth (Stacy 2017). Contamination of these environmental media can occur at each stage of UNGD (Saunders et al. 2016), and many air and water pollutants typically associated with UNGD sites have been identified as developmental and reproductive toxicants (Webb et al. 2014).

A number of studies examined UNGD and the potential health impacts without examining specific age groups such as children and adolescents. Recent papers reviewed all of the pertinent UNGD and health studies and reported the health effect categories that have been studied (McMullin et al. 2017; Stacy 2017). Generally, health effects studied thus far include: birth outcomes, birth defects, cancer, blood/immune, cardiovascular, gastrointestinal, musculoskeletal, neurological, psychological, and respiratory outcomes, and dermatological symptoms (McMullin et al. 2017). While some of these studies presented limited or mixed evidence for certain health impacts, no studies were found to have substantial or moderate evidence for health impacts, noting the need for higher quality studies to confirm or dispute the findings from these studies that have been predominantly hypothesis generating (McMullin et al. 2017).

Specific to child and adolescent health, several studies investigated birth outcomes (Casey et al. 2015; Hill 2012; Ma et al. 2016; McKenzie et al. 2014; Stacy et al. 2015). These studies found associations between UNGD activity and a range of outcomes, including preterm birth, low birth weight, small for gestational age, congenital heart defects, neural tube defects, and physician-recorded high risk pregnancy (Casey et al. 2015; Hill 2012; McKenzie et al. 2014; Stacy et al. 2015). One study examined childhood cancer, noting that all childhood cancers and childhood leukaemia were close to expected before and after UNGD with the exception of a slightly elevated standardised incidence ratio for central nervous system tumours (Fryzek et al. 2013). Another study considered children to some extent in its investigation of health outcomes using routinely collected data, such as emergency room and inpatient admissions (Coons and Walker 2008). Higher rates of certain respiratory outcomes (e.g., asthma) were noted for children living in areas with higher levels of UNGD.

Several studies have raised issues about children’s health and UNGD, including their vulnerability compared to adults, a lack of paediatric-specific data, the use of endocrine disrupting chemicals in the industry, and issues with UNGD development moving in closer proximity to schools and residential areas (Brown et al. 2015; Finkel et al. 2013; Kassotis et al. 2016, 2014; Lauver 2012; Webb et al. 2014; Witter et al. 2008). A recent review of potential water and air contaminants associated with UNGD concluded that more research is needed on UNGD and cancer risks, specifically for childhood leukaemia (Elliott et al. 2017). Finkel, Hays, and Law (2013) noted that “the extent of health risks associated with unconventional natural gas operations among children is unknown”.

Compared to adults, children have different exposures, absorption pathways, and responses to illness and the environment (Bearer 1995; Merrick 2013). These differences also vary throughout childhood, and there are periods (i.e., critical windows of exposure) where children are more susceptible to certain exposures (Agency for Toxic Substances and Disease Registry 2012; Bearer 1995). A number of chronic health outcomes in later life are associated with reduced foetal growth, demonstrating the importance of improving foetal health to improve health across a person’s lifespan (Barker 2007; Stacy 2017).

Children are considered a vulnerable, sensitive population. Hence, children and adolescents warrant separate examination for potential health impacts of CSG development. This was echoed by Webb et al. (2014), who concluded potential health impacts of UNGD on infants and children must be studied via rapid and thorough health investigations. Due to the lack of UNGD and health studies present when this study was designed and conducted, this study was exploratory and examined a range of health outcomes to generate hypotheses for future work that may relate to the putative exposures described above. Previous Australian studies found increased hospitalisation rates for neoplasms and blood/immune diseases for people living in CSG areas compared to a rural/agricultural area and increased hospitalisation rates for congenital anomalies for the CSG area compared to a coal mining area (Werner et al. 2016a, b). This exploratory analysis thus builds on those previous Australian studies to examine age-specific hospitalisation rates for children and adolescents aged 0–19 years old in a CSG study area compared to two other study areas (coal mining and rural/agricultural) in Queensland over the period 1995–2011.

## Methods

### Ethics Approval

Queensland Health granted access to confidential information (Approval Number RD004515) after ethics approvals were obtained from the University of Queensland Human Research Ethics Committee (Approval Number 2012000582).

### Study Location

**Fig. 1:**
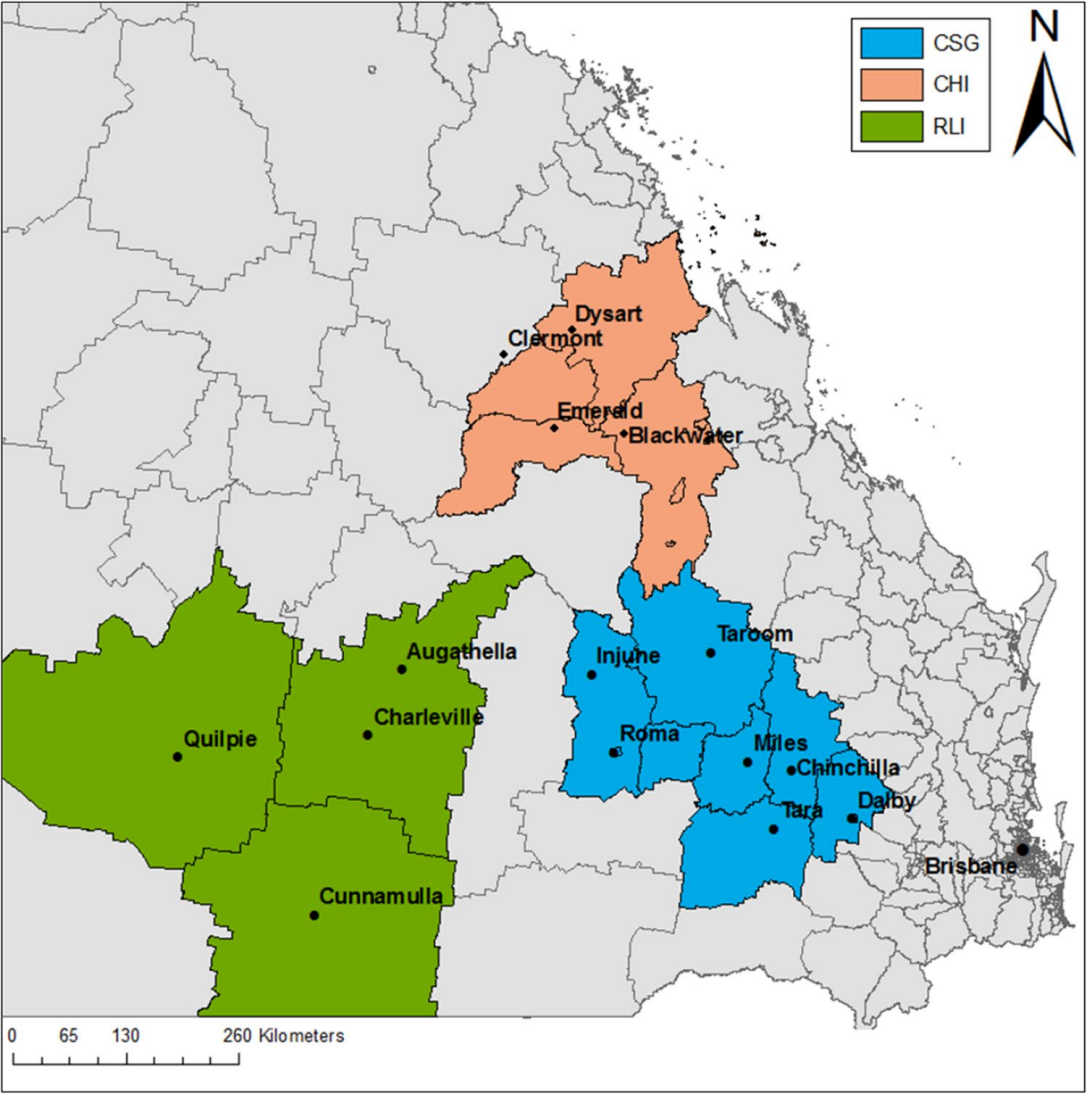
The coal seam gas (CSG), coal high-impact (CHI), and rural low-impact (RLI) study areas, grouped by SLAs within Queensland, Australia

The study was conducted across three rural geographic areas in Queensland, Australia, defined as: a CSG area, a coal high-impact (CHI) area, and a rural low-impact (RLI) area. Study areas were aggregates of statistical local areas (SLAs), as shown in Fig. 1. SLAs are the base geographic spatial units used to collect data in Australia (Australian Bureau of Statistics 2011).

The study areas were selected by examining CSG well and coal mine locations at the time of site selection (2011). The CHI area contained predominantly coal mining activity with minimal CSG activity, and the RLI area had no coal mining or CSG development at the time of site selection (Werner et al. 2016a). The CSG and CHI areas had similar populations (44,217 and 35,142 persons in 2011, respectively), while the RLI area had fewer people due to being a more regional area (7747 persons in 2011) (Australian Bureau of Statistics 2014; Werner et al. 2016a). Table 1 provides an overview of the sociodemographic characteristics for each study area across the study period.

**Table 1:** Sociodemographic characteristics for the coal seam gas, rural low-impact, and coal high-impact study areas across the study time period (Werner et al. 2016a, b)

	CSG			RLI			CHI		
	1995	2003	2011	1995	2003	2011	1995	2003	2011
Population	40,100	40,529	44,217	8804	8306	7747	32,508	30,644	35,142
Percentage male	51.45	51.50	51.76	52.77	51.70	51.00	53.83	53.82	53.97
Percentage female	48.55	48.50	48.24	47.23	48.30	49.00	46.17	46.18	46.03
Percentage Indigenous persons	2.83	4.22	4.86	10.99	13.14	15.64	5.24	4.84	5.71
Percentage persons Australian-born	89.35	86.74	83.40	92.77	87.21	83.36	89.77	86.11	77.78
Percentage persons employed full-time	31.39	31.09	31.86	33.79	33.27	33.12	36.23	37.04	36.24
Percentage persons in managerial, administrative, or professional occupations	14.90	15.61	15.30	13.72	14.79	16.28	9.84	11.47	12.05
Weighted average median weekly household income	485.81	749.24	1135.58	475.15	696.97	946.35	926.27	1250.16	2182.04
Weighted average mean household size	2.71	2.56	2.50	2.71	2.45	2.31	3.14	3.0	2.99

*CSG* coal seam gas, *RLI* rural low-impact, *CHI* coal high-impact

### Data

Details on the data used for this study have previously been described (Werner et al. 2016a). Hospital admission records were obtained from the Queensland Hospital Admitted Patient Data Collection (QHAPDC) for the period 1995–2011 for the three study areas. These data are put through extensive validation processes to ensure high quality, accurate data (Queensland Government 2012). Variable selection was based on the literature and putative confounders. Population data were obtained for each study area from the Australian Bureau of Statistics (ABS) and included estimated resident population (ERP) by age group (0–4 years, 5–9 years, 10–14 years, and 15–19 years), and gender for each calendar year. Covariate data (proportion Australian-born, proportion employed full-time, proportion Indigenous, proportion in white collar occupations, median household income, and mean household size) were obtained from the ABS, and were used to adjust for sociodemographic differences (Australian Bureau of Statistics 2014; Werner et al. 2016a).

### Analyses

Age-specific negative binomial regression models were used for all-cause hospitalisations and for each International Classification of Diseases (ICD) chapter, separately, for each age group within the child and adolescent cohort. Age-specific admission counts were modelled, offset by the log of the population, with time serving as a continuous ‘period’ variable. The outcome of primary interest was the interaction between area and period, which assessed the relative change in slope over time between the three study areas.

The fit of these models were assessed using goodness of fit criteria (Ismail and Jemain 2007; Zhang and Liu 2013), and rate ratios (RR; 95% CI) were calculated so that relative changes in the slope of age-specific hospitalisation rates could be identified in the CSG area relative to the CHI area and to the RLI area. Age-specific models were adjusted for gender and other covariates to provide adjusted RRs. The strongest associations were considered to be those associations where increasing rates were observed in the CSG area compared to rates in the CHI area *and* compared to rates in the RLI area. SAS 9.4 was used for the statistical analyses (SAS Institute, Cary NC, USA).

## Results

The CSG and CHI areas were more populous than the RLI area, and the CHI area had a slightly higher proportion of males to females. Additionally, the RLI area had a larger proportion of Indigenous persons and a lower average median weekly income compared to the CSG and CHI areas. Other sociodemographic characteristics are shown in Table 1.

**Fig. 2:**
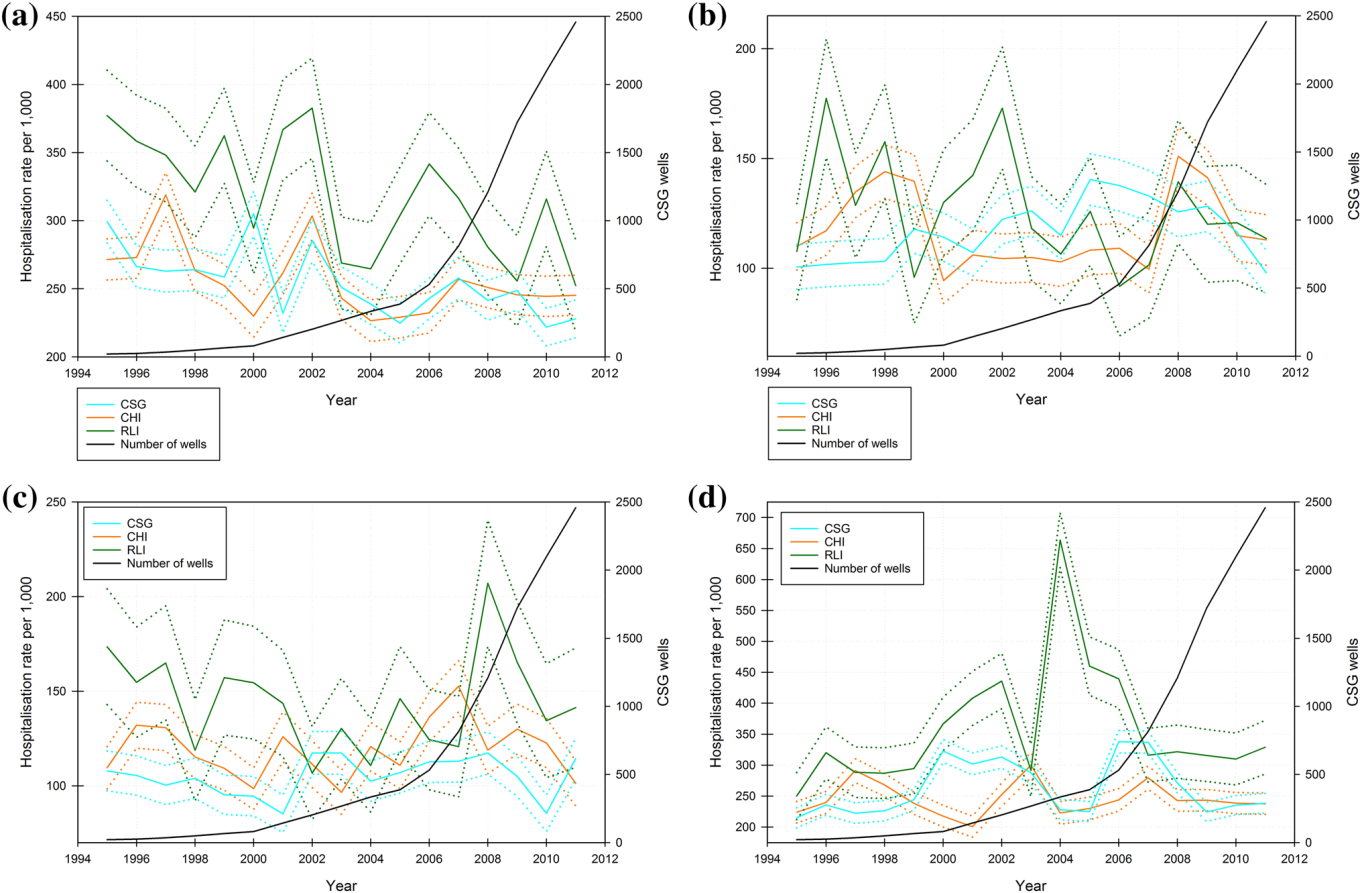
Age-specific, all-cause hospitalisation rates per 1000 persons for the CSG, CHI, and RLI areas, 1995–2011 for: **a** 0–4 years old; **b** 5–9 years old; **c** 10–14 years old; and **d** 15–19 years old

For the period 1995–2011, there were 80,882 child and adolescent hospital admissions in the three study areas, which comprised 17.6% of all admissions in these areas over this time period. Child and adolescent hospital admissions from the CSG, CHI, and RLI areas made up 46.7, 41.7, and 11.6% of these admissions, respectively. Changes over time in ‘All-cause’ admissions in the CSG area relative to the other two study areas, categorised by age group, are shown in Fig. 2, along with CSG well numbers during the same period.

The average annual rates of hospitalisation for each age group and study area are shown in Table 2. Higher rates were present in the 0–4 year old age group compared to other age groups for ‘Infectious disease’, ‘Ear’, and ‘Respiratory’ diseases whereas in the 15–19 year old age group, higher rates were observed for ‘Mental disorders’, ‘Genitourinary’ diseases, and ‘Injuries’.

**Table 2:** Average annual rate per 1000 persons for children and adolescents in each study area categorised by ICD chapters

	CSG	CHI	RLI		CSG	CHI	RLI
All-cause				Respiratory			
0–4 years	254.64	255.77	318.31	0–4 years	64.77	61.16	94.22
5–9 years	117.07	117.40	126.50	5–9 years	23.52	23.35	33.29
10–14 years	104.96	118.97	144.37	10–14 years	12.36	11.60	22.87
15–19 years	262.73	245.36	358.63	15–19 years	16.09	15.79	24.99
Infectious disease				Digestive			
0–4 years	21.69	22.13	33.96	0–4 years	21.16	18.30	16.70
5–9 years	6.98	5.97	9.12	5–9 years	16.34	17.04	14.92
10–14 years	4.03	3.67	9.77	10–14 years	11.27	13.22	9.54
15–19 years	7.34	5.73	10.81	15–19 years	31.05	32.65	34.28
Neoplasms				Skin			
0–4 years	1.96	2.76	1.70	0–4 years	3.35	5.49	7.80
5–9 years	2.83	1.25	0.51	5–9 years	2.69	3.42	4.57
10–14 years	3.24	2.87	1.12	10–14 years	2.93	5.04	7.58
15–19 years	3.80	4.21	1.94	15–19 years	7.46	8.77	14.72
Blood/immune				Musculoskeletal			
0–4 years	0.91	1.13	1.60	0–4 years	1.35	1.36	2.02
5–9 years	1.19	0.88	0.79	5–9 years	1.66	1.45	3.12
10–14 years	0.53	0.58	0.31	10–14 years	3.83	3.91	6.14
15–19 years	1.81	0.58	1.16	15–19 years	11.89	9.66	8.57
Endocrine				Genitourinary			
0–4 years	2.10	2.30	1.24	0–4 years	5.78	6.38	7.31
5–9 years	4.23	1.15	0.35	5–9 years	3.29	3.65	3.75
10–14 years	3.69	2.54	1.39	10–14 years	3.33	2.94	4.32
15–19 years	4.67	2.64	6.12	15–19 years	9.69	10.45	14.33
Mental disorders				Pregnancy			
0–4 years	0.33	0.16	0.33	10–14 years	0.18	0.36	0.41
5–9 years	0.55	0.25	0.35	15–19 years	42.64	37.82	53.47
				Perinatal			
10–14 years	2.30	1.96	2.45	0–4 years	35.30	32.01	28.53
15–19 years	12.70	8.02	14.12	5–9 years	0.02	0.00	0.00
Nervous system				Congenital			
0–4 years	5.21	3.55	3.26	0–4 years	12.16	9.68	10.82
5–9 years	3.34	1.64	3.23	5–9 years	2.43	1.90	1.67
10–14 years	2.54	1.57	4.03	10–14 years	1.85	1.37	1.94
15–19 years	3.58	3.13	6.08	15–19 years	1.15	1.20	1.67
Eye				Symptoms NEC			
0–4 years	2.36	2.23	2.31	0–4 years	18.36	19.40	28.29
5–9 years	1.13	0.98	1.21	5–9 years	6.25	7.23	7.74
10–14 years	0.68	0.66	2.01	10–14 years	7.67	8.18	12.40
15–19 years	0.72	1.03	1.90	15–19 years	14.58	14.79	26.42
Ear				Injuries			
0–4 years	10.82	12.93	14.63	0–4 years	30.70	32.91	39.63
5–9 years	6.42	9.23	7.42	5–9 years	24.78	30.20	26.64
10–14 years	1.84	2.66	4.55	10–14 years	34.84	44.70	46.55
15–19 years	0.73	1.23	1.29	15–19 years	60.14	70.04	88.74
Circulatory							
0–4 years	0.65	0.75	0.79				
5–9 years	1.17	0.72	1.00				
10–14 years	1.37	0.97	1.27				
15–19 years	1.53	2.04	2.14				

*CSG* coal seam gas, *CHI* coal high-impact, and *RLI* rural low-impact

ICD code ranges are: A00–B99 = ‘Certain infectious and parasitic diseases’; C00–D48 = ‘Neoplasms’; D50–D89 = ‘Diseases of the blood and blood-forming organs and certain disorders involving the immune mechanism’; E00–E90 = ‘Endocrine, nutritional and metabolic diseases’; F00–F99 = ‘Mental and behavioural disorders’; G00–G99 = ‘Diseases of the nervous system’; H00–H59 = ‘Diseases of the eye and adnexa’; H60–H95 = ‘Diseases of the ear and mastoid process’; I00–I99 = ‘Diseases of the circulatory system’; J00–J99 = ‘Diseases of the respiratory system’; K00–K93 = ‘Diseases of the digestive system’; L00–L99 = ‘Diseases of the skin and subcutaneous tissue’; M00–M99 = ‘Diseases of the musculoskeletal system and connective tissue’; N00–N99 = ‘Diseases of the genitourinary system’; O00–O99 = ‘Pregnancy, childbirth and the puerperium’; P00–P96 = ‘Certain conditions originating in the perinatal period’; Q00–Q99 = ‘Congenital malformations, deformations and chromosomal abnormalities’; R00–R99 = ‘Symptoms, signs and abnormal clinical and laboratory findings, not elsewhere classified’; and S00–T98 = ‘Injury, poisoning and certain other consequences of external causes’

Tables 3 and 4 show the results for the adjusted models for ‘All-cause’ admissions and all ICD chapters, separated by age group. The results are summarised by each age group below.

**Table 3:** Adjusted rate ratios (RR) and 95% CI for hospitalisation rates over time (All-cause and ICD Chaps. 1–9) in three study areas for a child and adolescent cohort

	CSG vs. CHI	CSG vs. RLI	CHI vs. RLI
All-cause			
0–4 years	1.00 [0.98, 1.03]	1.02 [0.99, 1.05]	1.01 [0.98, 1.05]
5–9 years	1.04 [1.00, 1.09]	1.09 [1.04, 1.14]	1.04 [0.99, 1.09]
10–14 years	0.98 [0.94, 1.02]	1.03 [0.98, 1.08]	1.05 [1.00, 1.11]
15–19 years	1.06 [1.00, 1.12]	1.05 [0.99, 1.12]	0.99 [0.93, 1.06]
Infectious disease			
0–4 years	0.98 [0.91, 1.07]	1.05 [0.95, 1.15]	1.07 [0.96, 1.18]
5–9 years	1.08 [0.98, 1.20]	1.12 [1.00, 1.25]	1.03 [0.91, 1.17]
10–14 years	0.96 [0.84, 1.09]	1.07 [0.93, 1.24]	1.12 [0.95, 1.33]
15–19 years	1.12 [0.99, 1.27]	1.04 [0.91, 1.18]	0.93 [0.80, 1.08]
Neoplasms			
0–4 years	0.87 [0.67, 1.13]	0.85 [0.60, 1.19]	0.97 [0.69, 1.38]
5–9 years	1.00 [0.68, 1.49]	1.95 [1.27, 3.00]	1.94 [1.19, 3.15]
10–14 years	0.89 [0.69, 1.15]	1.22 [0.87, 1.71]	1.37 [0.98, 1.92]
15–19 years	1.05 [0.95, 1.17]	1.18 [1.04, 1.33]	1.12 [0.98, 1.28]
Blood/immune			
0–4 years	0.96 [0.67, 1.37]	1.23 [0.82, 1.83]	1.28 [0.82, 2.00]
5–9 years	0.95 [0.33, 2.77]	5.67 [2.39, 13.44]	5.96 [1.52, 23.46]
15–19 years	0.61 [0.38, 0.98]	0.86 [0.56, 1.31]	1.40 [0.80, 2.46]
Endocrine			
0–4 years	1.07 [0.88, 1.30]	1.25 [0.99, 1.57]	1.16 [0.91, 1.49]
5–9 years	1.03 [0.73, 1.44]	1.03 [0.71, 1.47]	1.00 [0.65, 1.54]
10–14 years	0.75 [0.58, 0.95]	0.66 [0.51, 0.85]	0.88 [0.65, 1.20]
15–19 years	0.66 [0.51, 0.86]	0.75 [0.60, 0.95]	1.13 [0.82, 1.56]
Mental disorders			
10–14 years	0.87 [0.70, 1.08]	0.96 [0.74, 1.23]	1.10 [0.84, 1.44]
15–19 years	0.95 [0.84, 1.07]	1.17 [1.04, 1.32]	1.24 [1.07, 1.44]
Nervous system			
0–4 years	1.08 [0.91, 1.27]	0.91 [0.76, 1.09]	0.84 [0.68, 1.04]
5–9 years	1.02 [0.83, 1.25]	0.99 [0.81, 1.21]	0.97 [0.75, 1.26]
10–14 years	0.86 [0.69, 1.07]	1.12 [0.89, 1.40]	1.31 [0.99, 1.73]
15–19 years	1.05 [0.85, 1.29]	0.90 [0.71, 1.13]	0.85 [0.67, 1.09]
Eye			
15–19 years	1.17 [0.82, 1.68]	1.09 [0.73, 1.64]	0.93 [0.60, 1.44]
Ear			
0–4 years	1.04 [0.97, 1.11]	1.00 [0.92, 1.09]	0.96 [0.89, 1.05]
5–9 years	1.07 [0.97, 1.19]	1.12 [0.99, 1.27]	1.04 [0.92, 1.19]
10–14 years	1.02 [0.87, 1.20]	1.03 [0.85, 1.24]	1.01 [0.83, 1.22]
Circulatory			
0–4 years	0.71 [0.51, 0.98]	0.81 [0.55, 1.19]	1.13 [0.76, 1.70]
5–9 years	1.13 [0.84, 1.54]	0.91 [0.65, 1.26]	0.80 [0.55, 1.16]
15–19 years	1.02 [0.81, 1.27]	0.96 [0.73, 1.27]	0.95 [0.71, 1.26]

CSG is compared against the CHI reference (Column 1) and the RLI reference (Column 2). CHI is compared against the RLI reference (Column 3). *CHI* coal high-impact, *CSG* coal seam gas, and *RLI* rural low-impact

Adjusted for: gender, proportion Australian-born, proportion employed full-time, proportion Indigenous, proportion white collar, weight average median household income, and weight average mean household size

ICD chapter code ranges are shown in Table 1. ‘Blood/immune’ 10–14 years, ‘Mental disorders’ 0–4 years and 5–9 years, ‘Eye’ 0–4 years, 5–9 years, and 10–14 years, ‘Ear’ 15–19 years, and ‘Circulatory’ 10–14 years excluded from table due to an insufficient number of cases

**Table 4:** Adjusted rate ratios (RR) and 95% CI for hospitalisation rates over time (ICD Chaps. 10–19) in three study areas for a child and adolescent cohort

	CSG vs. CHI	CSG vs. RLI	CHI vs. RLI
Respiratory			
0–4 years	1.07 [1.04, 1.11]	1.06 [1.02, 1.10]	0.99 [0.94, 1.03]
10–14 years	1.09 [1.01, 1.18]	1.11 [1.01, 1.21]	1.01 [0.92, 1.11]
15–19 years	1.00 [0.92, 1.08]	1.00 [0.92, 1.09]	1.00 [0.91, 1.11]
Digestive			
0–4 years	1.00 [0.95, 1.06]	1.03 [0.97, 1.11]	1.03 [0.96, 1.11]
5–9 years	1.04 [0.96, 1.12]	0.99 [0.91, 1.08]	0.95 [0.87, 1.05]
10–14 years	1.07 [0.99, 1.17]	1.19 [1.07, 1.31]	1.11 [0.99, 1.23]
15–19 years	1.08 [1.02, 1.14]	1.00 [0.94, 1.07]	0.93 [0.87, 1.00]
Skin			
0–4 years	0.98 [0.88, 1.09]	1.02 [0.89, 1.16]	1.03 [0.91, 1.18]
5–9 years	0.91 [0.80, 1.05]	1.10 [0.93, 1.30]	1.20 [1.01, 1.43]
10–14 years	0.97 [0.86, 1.10]	0.89 [0.76, 1.04]	0.92 [0.79, 1.07]
15–19 years	1.10 [0.98, 1.22]	1.00 [0.88, 1.13]	0.91 [0.79, 1.04]
Musculoskeletal			
0–4 years	0.97 [0.74, 1.26]	0.78 [0.56, 1.08]	0.80 [0.56, 1.15]
5–9 years	1.13 [0.87, 1.45]	1.36 [1.03, 1.81]	1.21 [0.88, 1.66]
10–14 years	1.05 [0.90, 1.21]	1.06 [0.90, 1.25]	1.01 [0.84, 1.21]
Genitourinary			
0–4 years	0.99 [0.89, 1.09]	0.97 [0.85, 1.10]	0.98 [0.86, 1.11]
5–9 years	1.10 [0.93, 1.29]	1.16 [0.95, 1.42]	1.06 [0.87, 1.29]
10–14 years	1.14 [0.97, 1.34]	1.18 [0.99, 1.41]	1.04 [0.85, 1.26]
15–19 years	0.99 [0.91, 1.09]	1.00 [0.90, 1.10]	1.00 [0.90, 1.12]
Pregnancy			
15–19 years	1.02 [0.96, 1.08]	1.05 [0.98, 1.12]	1.03 [0.96, 1.11]
Perinatal			
0–4 years	0.89 [0.84, 0.93]	0.94 [0.89, 1.00]	1.06 [1.00, 1.13]
Congenital			
0–4 years	1.08 [0.99, 1.17]	1.12 [1.02, 1.23]	1.04 [0.94, 1.15]
5–9 years	1.18 [0.99, 1.41]	0.95 [0.78, 1.16]	0.80 [0.65, 0.99]
10–14 years	1.07 [0.85, 1.34]	0.93 [0.73, 1.17]	0.87 [0.66, 1.13]
15–19 years	0.84 [0.60, 1.16]	0.87 [0.62, 1.22]	1.04 [0.71, 1.54]
Symptoms NEC			
0–4 years	0.93 [0.86, 1.01]	0.99 [0.90, 1.08]	1.06 [0.96, 1.17]
10–14 years	0.95 [0.86, 1.05]	1.00 [0.89, 1.13]	1.05 [0.93, 1.20]
15–19 years	0.94 [0.86, 1.04]	0.91 [0.82, 1.02]	0.97 [0.86, 1.09]
Injuries			
0–4 years	1.03 [0.97, 1.10]	1.00 [0.93, 1.07]	0.97 [0.90, 1.04]
5–9 years	1.01 [0.96, 1.07]	1.08 [1.01, 1.15]	1.06 [0.99, 1.13]
10–14 years	0.98 [0.93, 1.04]	1.02 [0.96, 1.09]	1.04 [0.97, 1.12]
15–19 years	1.06 [1.00, 1.11]	1.03 [0.97, 1.10]	0.98 [0.92, 1.05]

CSG is compared against the CHI reference (Column 1) and the RLI reference (Column 2). CHI is compared against the RLI reference (Column 3). *CHI* coal high-impact, *CSG* coal seam gas, and *RLI* rural low-impact

Adjusted for: gender, proportion Australian-born, proportion employed full-time, proportion Indigenous, proportion white collar, weight average median household income, and weight average mean household size

ICD chapter code ranges are shown in Table 1. ‘Respiratory’ 5–9 years, ‘Musculoskeletal’ 15–19 years, ‘Pregnancy’ 0–4 years, 5–9 years, and 10–14 years, ‘Perinatal’ 5–9 years, 10–14 years, and 15–19 years, and ‘Symptoms NEC’ 5–9 years excluded from table due to an insufficient number of cases

### 0–4 year olds

As shown in Table 4, ‘Respiratory’ disease-related admission rates increased in the CSG area relative to *both* the CHI and RLI areas. Rates in the CSG area increased by 7% per year compared to the CHI area and by 6% per year compared to the RLI area. For this age group, the highest proportion of ‘Respiratory’ disease admissions fell under the ‘Acute upper respiratory infections’ subchapter. ‘Other acute lower respiratory infections’, ‘Influenza and pneumonia’, and ‘Chronic lower respiratory diseases’ accounted for the second through fourth subchapters with the most ‘Respiratory’ disease admissions. For the CSG area, primary diagnoses were predominantly for ‘Acute upper respiratory infection, unspecified’, ‘Acute obstructive laryngitis’, ‘Pneumonia, unspecified’, and ‘Asthma, unspecified’.

Additionally, ‘Congenital’ malformation admission rates increased by 12% per year in the CSG area compared to the RLI area (Table 4).

### 5–9 year olds

The strongest age-specific effects were observed in this age group. ‘All-cause’ hospitalisation rates increased by 4% per year in the CSG area relative to the CHI area and by 9% per year in the CSG area relative to the RLI area, after adjusting for relevant variables (Table 3).

As shown in Table 3, there was a 467% per year increase in ‘Blood/immune’ disease admission rates in the CSG area per year relative to the RLI area, after adjustment. For 5–9 year olds in the CSG area, the most common ‘Blood/immune’ diseases were ‘Agranulocytosis’, ‘Secondary thrombocytopenia’, and ‘Anaemia, unspecified’. Over the entire study period, the total number of ‘Blood/immune’ disease admissions were 67, 46, and 10 for the CSG, CHI, and RLI areas, respectively.

Adjustment for covariates revealed a 95% increase per year in ‘Neoplasms’ admission rates in the CSG area relative to the RLI area. The CHI area also showed an increase of 94% per year relative to the RLI area. ‘Musculoskeletal’ disease admission rates and ‘Injuries’ admission rates increased by 36% per year and 8% per year, respectively, in the CSG area compared to the RLI area (Table 4).

### 10–14 year olds

Adjusted model results showed that ‘Respiratory’ disease-related rates increased by 9 and 11% per year in the CSG area relative to the CHI area and to the RLI area, respectively (Table 4). For all three study areas, the greatest proportions of ‘Respiratory’ disease admissions came from the ‘Other diseases of upper respiratory tract’ subchapter, followed by the ‘Acute upper respiratory infections’, ‘Chronic lower respiratory diseases’, and ‘Influenza and pneumonia’ subchapters. In the CSG area, the most common diagnoses in the ‘Respiratory’ chapter, which differ from the most common diagnoses for 0–4 year olds, were ‘Chronic tonsillitis’, ‘Asthma, unspecified’, and ‘Acute tonsillitis’.

Additionally, Table 4 shows the adjusted model results indicated that *‘Digestive’* disease rates increased by 19% in the CSG area compared to the RLI area.

### 15–19 year olds

The adjusted model results showed admission rates due to any cause increased by 6% per year in the CSG area compared to the CHI area. For this age group, ‘Neoplasm’-related admission rates increased by 18% per year in the CSG area relative to the RLI area (Table 3). After adjusting for covariates, ‘Mental disorders’-related admission rates increased by 17% per year in the CSG area compared to the RLI area. These admission rates also increased by 24% in the CHI area relative to the RLI area. Finally, ‘Digestive’ disease hospitalisation rates increased by 8% per year in the CSG area compared to the CHI area (Table 4).

## Discussion

Increasing rates of child and adolescent hospital admissions in the CSG area were present when compared to child and adolescent hospital admissions in the CHI and/or RLI areas for a number of ICD chapters This is the first study in Australia to examine CSG development and hospitalisation rates for children and adolescents.

In a previous study of all-ages hospital admissions in the same geographic areas, the strongest associations in terms of relative changes in hospital admissions were evident for ‘Neoplasms’ and ‘Blood/immune’ diseases in the CSG area compared to the RLI area and for ‘Congenital’ outcomes in the CSG area compared to the CHI area (Werner et al. 2016a, b). In the current study, the strongest associations were observed for ‘All-cause’ admission rates for 5–9 year olds and ‘Respiratory’ disease admission rates for 0–4 and 10–14 year olds. For both of these outcomes, rates in the CSG area increased compared to rates in the CHI area *and* compared to rates in the RLI area.

As previously noted, the majority of the UNGD literature covers shale gas development in the USA (Werner et al. 2015). It has been stated that the outcomes from these studies may not necessarily translate to the CSG context due to a number of differences, including regulatory aspects, geological formations, and extraction processes (NSW Chief Scientist and Engineer 2013; Werner et al. 2016a). However, due to the lack of available CSG-specific literature, the UNGD evidence base is discussed below.

While not particularly focused on children and adolescents, numerous outcomes have been identified in the literature as being potentially positively associated with UNGD including: infectious disease outcomes (Werner et al., n.d.; Zou et al. 2006); mental health (Ferrar et al. 2013; McDermott-Levy and Garcia 2016; Morgan et al. 2016; Perry 2013; Steinzor et al. 2013; Subra 2009; Werner et al., n.d.); neoplasms (McKenzie et al. 2012; Werner et al., n.d., 2016a); blood/immune diseases (Werner et al., n.d., 2016a); birth outcomes (Hill 2012; McKenzie et al. 2014; Stacy et al. 2015); cardiovascular outcomes (Subra 2010); dermatological symptoms (Rabinowitz et al. 2015; Steinzor et al. 2013; Subra 2009); eye symptoms (Steinzor et al. 2013; Subra 2010); ear/nose/mouth/throat symptoms (Steinzor et al. 2013; Subra 2009, 2010); gastrointestinal outcomes (Ferrar et al. 2013; Steinzor et al. 2013); musculoskeletal symptoms (Steinzor et al. 2013; Subra 2009); neurological symptoms (Subra 2010); and respiratory outcomes (Brown et al. 2015; Rabinowitz et al. 2015; Steinzor et al. 2013; Subra 2009). Most of these outcomes have been discussed in the context of symptoms reported by residents living near UNGD rather than residents being hospitalised for such conditions.

The majority of studies have not specifically examined children; however, a small number of studies have examined children’s health in relation to UNGD. These studies have examined birth outcomes (Coons and Walker 2008; Hill 2012; McKenzie et al. 2014; Stacy et al. 2015) or childhood cancer (Fryzek et al. 2013) in the USA. Potential associations were observed between UNGD and certain birth outcomes (e.g., congenital heart defects, low birth weight, neural tube defects, and small for gestational age). Such outcomes would fall under the broader ‘Perinatal’ or ‘Congenital’ ICD chapters examined here; however, the aforementioned studies used specific datasets, such as data from vital statistics or birth defects monitoring databases, as opposed to primary diagnosis codes from hospital admissions databases.

Only one study used hospital admissions data and specifically examined a child and adolescent cohort (Coons and Walker 2008). Those authors found that Garfield County (i.e., the county with UNGD) had the highest rate for upper respiratory infections, bronchitis, and asthma in children as compared to three other counties, but the lowest rates for other respiratory infections or inflammation in children. In our study, 0–4 and 10–14 year olds in the RLI area had the highest rate of ‘Respiratory’ disease-related admissions, showing a greater absolute risk; however, after accounting for sociodemographic variables, the CSG area had increasing rates compared to the CHI and RLI areas, showing a greater relative risk. ‘Acute upper respiratory infections’ and ‘Asthma’ were some of the primary diagnoses with the greatest number of admissions for 0–4 and/or 10–14 year olds, which are similar to the diagnoses found for Garfield County.

Webb et al. (2014) reviewed the literature to examine developmental and reproductive effects of chemicals potentially associated with UNGD and noted that inhalation is a common route of exposure. Air quality can be affected by UNGD as a variety of chemicals are emitted including methane, nitrogen oxides (NO_x_), and volatile organic compounds (VOCs) (Webb et al. 2014). Ground-level ozone, which can create additional potential health impacts, is formed through NO_x_ reacting with VOCs and sunlight (Webb et al. 2014). While exposure to these pollutants could contribute to respiratory-related outcomes, Queensland Health noted that reported concentrations of numerous atmospheric analytes measured in a CSG area (i.e., Tara, Queensland) were not expected to be associated with adverse health impacts (Queensland Government 2013). However, it was also noted that an air quality monitoring program could be put in place to analyse ambient air quality in CSG development areas in Queensland to better understand these associations, if any (Queensland Government 2013).

Coons and Walker (2008) also noted that ‘Blood/immune’ disease admission rates for Garfield County decreased over the 7-year study period. This contradicts the findings presented in the current study and those findings presented in previous studies (Werner et al., 2016a). However, while the largest effect sizes were noted for ‘Blood/immune’ diseases for 5–9 year olds in the CSG and CHI areas relative to the RLI area, the ‘Blood/immune’ disease effect sizes must be considered alongside the average annual rates that were presented. The average rates for these age groups ranged from 0.79 to 1.19 per 1000 persons for the three study areas, indicating a low base rate where small numbers would likely contribute to larger estimates and wider confidence intervals.

In comparing the findings presented here to findings from a previous Australian study that examined age-specific impacts on younger, middle, and older adults (Werner et al., n.d.), several key differences appeared. ‘Congenital’ outcomes were found only for the 0–4 year olds, which would be expected due to the nature of the health outcomes within the ICD chapter. ‘Respiratory’ and ‘Digestive’ disease-related rate increases were found only in the child and adolescent cohort, and ‘Musculoskeletal’ and ‘Injuries’ rate increases were observed only in the 5–9 year old age group. Increases were noted for several ICD chapters that spanned the child and adolescent and adult cohorts including ‘Neoplasms’, ‘Blood/immune’ diseases, and ‘Mental disorders’. Therefore, the results presented here suggest that there are age-specific health impacts that need to be considered, particularly given the uniqueness and vulnerability of children (Merrick 2013).

Several important limitations are associated with this study (Werner et al. 2016a). These include the use of hospital admissions data, which represents more severe forms of morbidity, and excludes those residents who do not seek health care, meaning that the rates presented here have likely been underestimated. The coverage of the QHAPDC dataset is extensive, covering all hospitals that are permitted to admit patients, including public hospitals, licensed private hospitals, day surgery units, and public psychiatric hospitals (Queensland Health 2015). However, these data do not include less severe sequelae for which a person is not admitted to hospital. Outcomes and syndromes that fall in this category may be seen by a GP or not at all, so would not be captured in these data. Additionally, repeat admissions were included in the dataset, meaning that a resident could have been admitted on more than one occasion in 1 year for the same primary diagnosis.

The nature of this study and the available data means that critical periods, other than the period effects of CSG development, such as in utero exposures were not considered. This study only assesses exposures and outcomes during the study period. Using very broad measures of exposure, it should be noted that the oldest age group (15–19 year olds) would not have been exposed throughout their lives due to the timeline of CSG development. Furthermore, there are low case numbers for certain age groups and outcomes, so some associations that were found may be due to small numbers or stochastic variations. It is also possible that residual confounding or unmeasured confounding influenced the results (Fewell et al. 2007).

The results from this exploratory study indicate possible age-specific trends for potential health impacts of CSG within a child and adolescent cohort in Queensland, Australia that warrant further exploration with more sophisticated study designs. This study was not intended to examine potential causal associations. For the current study, data were grouped according to their respective broad geographic areas due to privacy and confidentiality concerns associated with data access and approval through Queensland Health, which also includes issues with potentially smaller numbers in the RLI area.

A future direction to consider is to look at sex-specific differences in the relationships presented here. Future studies should strive to use higher resolution data, if accessible. This would allow for additional research, such as spatial analyses to determine child and adolescent locations relative to home or school and areas where CSG well development is occurring. Additionally, specific datasets (e.g., congenital anomalies and perinatal outcomes) may be more useful to examine such outcomes more in-depth, as other studies have done elsewhere, as opposed to using a hospital admissions database. If similar assessments of health impacts were to continue, whether through the public or the private sector, this would ensure that stakeholders in the resources sector and communities would have an evidence-base to inform ongoing monitoring and evaluation.

These findings show that there are potential maternal and child health policy implications to consider in relation to CSG development activity. Additional work is needed to confirm the associations presented here and in other studies, but the current conclusions suggest the need for the consideration of the impacts of UNG activities in relation to the location of communities in which the activities occur (i.e., that there are appropriate setbacks based on health-related standards). Environmental public health practitioners should be included and consulted throughout the process. Governments should establish monitoring systems to allow for tracking the incidence and prevalence of diseases thought to be associated with UNGD (Finkel et al. 2013) in conjunction with exposure data, particularly for those exposures that occur in utero and may influence birth outcomes.

In conclusion, increasing rates of hospitalisation for children and adolescents were evident in the CSG area for certain ICD chapters compared to the rates in the CHI and/or RLI study areas. The results suggest that potential age-specific health impacts should be taken into account when considering current and future CSG development, as some trends were found for certain age groups and health outcomes. The findings suggest areas that should be explored further, including sub-chapters within specific ICD chapters and examining additional datasets for certain outcomes.

## Abbreviations

ABS: Australian Bureau of Statistics
CI: Confidence interval
CSG: Coal seam gas
CHI: Coal high-impact
DRG: Diagnostic-related grouping
ERP: Estimated resident population
ICD: International Classification of Diseases
RLI: Rural low-impact
SLA: Statistical local area
QHAPDC: Queensland Hospital Admitted Patient Data Collection
UNGD: Unconventional natural gas development
